# Potential role of hypothalamic microRNAs in regulation of FOS and FTO expression in response to hypoglycemia

**DOI:** 10.1007/s12576-019-00718-0

**Published:** 2019-11-14

**Authors:** Bashair M. Mussa, Jalal Taneera, Abdul Khader Mohammed, Ankita Srivastava, Debasmita Mukhopadhyay, Nabil Sulaiman

**Affiliations:** 1 Basic Medical Science Department, College of Medicine, University of Sharjah, P.O. Box: 27272, Sharjah, United Arab Emirates; 2 Basic Medical Science Department, College of Medicine, Sharjah Institute for Medical Research, University of Sharjah, P.O. Box: 27272, Sharjah, United Arab Emirates; 3 Sharjah Institute for Medical Research, University of Sharjah, P.O. Box: 27272, Sharjah, United Arab Emirates; 4 Family Medicine and Behavioral Science, College of Medicine, University of Sharjah, P.O. Box: 27272, Sharjah, United Arab Emirates

**Keywords:** FOS, FTO, microRNAs, Diabetes mellitus, Hypoglycemia-associated autonomic failure

## Abstract

Hypoglycemia-associated autonomic failure (HAAF) is a serious complication of diabetes which is associated with the absence of physiological homeostatic counter-regulatory mechanisms that are controlled by the hypothalamus and sympathetic nervous system. Identification of biomarkers for early detection of HAAF requires an advanced understanding of molecular signature of hypoglycemia which is yet to be identified. The outcomes of the present study have shown that the viability and the apoptotic rate of the hypothalamic neurons (mHypoE-N39) were decreased significantly due to hypoglycemia in a dose-dependent fashion (*p* < 0.05). Although there are more than 1000 miRNAs differentially expressed in hypothalamus, only twelve miRNAs (miR-7a, miR-7b, miR-9, miR-29b, miR-29c, miR-30a, miR-30b, miR-30c, miR-101b-3p, miR-181a-5p, miR-378-3p and miR-873-5p) were correlated to two main hypothalamic regulatory proteins, FOS and FTO. Expression of these proteins was very sensitive to hypoglycemia. We demonstrated that hypoglycemia modulates the expression of hypothalamic miRNAs that are related to FOS and FTO.

## Introduction

Hypothalamus is composed of multiple clusters of neurons which express different hormones, neurotransmitters and proteins. The latter are involved in regulation of a wide range of homeostatic processes, including cognitive functions, glucose sensing and metabolism [1, 2].

Interestingly, the functions of the hypothalamus evolve through lifetime to adapt to several external factors including environment, stress and lifestyle [3]. It has been found that aging, exposure to high-fat diet and stressful events influence the hypothalamic functions, dramatically [4, 5]. In addition, more studies have demonstrated that the threshold of the hypothalamic response is changeable, for instance, the response of leptin threshold to weight change [6].

The metabolic processes in the brain are mainly dependent on the peripheral glucose and any reduction in glucose beyond the physiological levels leads to activation of robust counter-regulatory response (CRR) [7]. The latter involves a group of neurons within the hypothalamus and the rostral ventro-lateral medulla which activate the sympathetic system that, in turn, stimulates adreno-medullary response leading to hypoglycemia awareness [8, 9]. Reduction or absence of CRR leads to hypoglycemia-associated autonomic failure (HAAF) [10]. Given that in diabetes mellitus (DM) the responses of the pancreatic islets to hypoglycemia are disabled, the body mainly depends on autonomic-adreno-medullary regulatory system to correct the hypoglycemia [11].

HAAF is mainly caused by the use of anti-diabetic medications, in particular, insulin-based treatments, with prevalence of 25% in patients with type 1 DM [12]. The consequences of HAAF range from confusion to convulsions, coma and even death and it also represents a major limitation to achieve an optimal control of DM and this influences the quality of life of patients with DM, adversely [13].

MicroRNAs (miRNAs), non-coding ribonucleic acid (RNA) molecules, negatively regulate the expression of various proteins within the hypothalamus [14]. MiRNAs mediate post-transcriptional silencing of target genes via binding to the 3′-untranslated regions (3′-UTR) of target mRNAs which eventually leads to inhibition of protein synthesis [14]. Hypothalamic miRNAs are involved in control of food intake and energy balance and their dysregulation in early life is associated with insulin resistance in adulthood [15]. It is believed that miRNAs are involved in regulation of metabolic profile via direct interaction with the regulatory hypothalamic proteins [16].

Previous reports have demonstrated changes in the expression of the hypothalamic miRNA after chronic hyperosmolar stimulation which, in turn, influenced the regulation of FOS [17]. In addition, FOS expression has been shown to decrease within the medial and dorsal hypothalamic areas in response to hypoglycemia [18–22]. Given that these hypothalamic areas are involved in the CRR, it was hypothesized that the reduction in FOS expression is an indicator of HAAF [23]. On the other hand, changes in FOS expression in diabetic neuropathy and other stressful events including hypoxia–ischemia suggest a neuro-protective role for FOS expression [24, 25]. Moreover, it has been reported that FOS is involved in regulation of food intake and gastric motility [26]. Therefore, it seems that the involvement of FOS in regulation of the neuronal signal is more complex than previously believed.

Fat mass and obesity-associated protein (FTO) is another protein that is highly expressed in the hypothalamic region with critical roles for control of energy balance in a nutritionally dependent manner [27]. Clinical studies have demonstrated a strong association between a single-nucleotide polymorphism in the FTO gene and obesity in diverse ethnic populations [28, 29]. In addition to its association with body mass index, variations in the FTO gene have been linked with glucose metabolism and type 2 DM [28, 30]. FTO mRNA is expressed in several tissue types, with most abundant expression in the hypothalamic nuclei, and is involved in regulation of several feeding processes [27, 28].

Taking into account the previous findings, it is crucial to investigate the molecule signature of hypoglycemia and the expression of hypothalamic miRNAs, FOS and FTO simultaneously to propose novel biomarkers for HAAF.

## Materials and methods

### Cells and culture conditions

Embryonic mouse hypothalamus cells (EHNs, mHypoE-N39) were obtained from Cedarlane (Burlington, Ontario, Canada) and cultured in Dulbecco's Modified Eagle's Medium (DMEM; Sigma-Aldrich) containing 4500 mg/L glucose supplemented with 10% Fetal Bovine Serum (FBS; Sigma-Aldrich), 1% penicillin/streptomycin (100 units/mL; Sigma-Aldrich) and maintained at 37 °C with humidified atmosphere of 5% CO_2_ and 95% air. The cells were cultured in 75 cm^2^ cell culture flask (Sigma-Aldrich). Four different conditions of low glucose were established by seeding the cells in DMEM containing 2000 mg/L, 900 mg/L, 500 mg/L and 200 mg/L of glucose. The low glucose conditions (2000 mg/L glucose) constituted of 44.4 mL of DMEM containing 4500 mg/L glucose, and 55.6 mL of DMEM without glucose. Similarly, low glucose conditions of 900 mg/L, 500 mg/L and 200 mg/L were obtained by mixing 20 mL, 11.2 mL and 4.44 mL of DMEM containing 4500 mg/L glucose, and 80 mL, 88.88 mL and 96.4 mL of DMEM without glucose, respectively. Cells were maintained under these regimens for 72 h; medium was changed every 48 h.

### Cell viability

Cell viability was measured using the 3-[4,5-dimethylthiazol-2-yl]-2,5diphenyltetrazolium bromide (MTT; Sigma-Aldrich) colorimetric assay. Briefly, 5000 cells were seeded in 96-well plates with 150 μL culture medium containing different glucose concentrations (2000, 900, 500, 200 mg/L of glucose) and maintained for 24, 48 and 72 h. The media were replaced with 100 μL of phosphate-buffered saline (PBS; Sigma-Aldrich) and 20 μL of MTT solution (5 mg/mL) and incubated at 37 °C for 180 min. MTT formzan crystals were dissolved in 100 μL of dimethyl sulfoxide (DMSO; Sigma-Aldrich) and absorbance was recorded at 570 nm on a microplate reader. The percentage of cell viability was calculated from the average 570 nm absorbance value as per the following equation: % cell viability = (OD 570 nm of sample/OD 570 nm of control) × 100.

### Apoptosis analysis

For apoptosis analysis, 1 × 10^6^ cells were seeded in a 6-well plate and maintained in different glucose concentrations for 72 h. Apoptosis assay was performed using annexin V-FITC apoptosis staining/detection kit (Abcam). Cells were harvested and washed twice with PBS. Around 0.5 × 10^5^ cells were resuspended in 500 µL of 1× binding buffer followed by adding 3 µL of Annexin V-FITC and 3 µL propidium iodide and incubated in dark for 10 min at room temperature. Cells were analyzed by BD Accuri flow cytometer (Becton Dickinson) applying FITC channel (515 nm) and PE detector (585 nm) and a minimum of 15,000 events were collected for the analysis. Compensation was performed using the single color controls with Annexin FITC and PI.

### Total RNA, miRNA extraction and cDNA synthesis

In a 6-well plate, 1.5 × 10^5^ cells were seeded and maintained in different glucose concentrations for 72 h. The total RNA was extracted using Pure Link RNA Mini Kit (Invitrogen) following the manufacturer’s instruction. MiRNA was extracted using miRNA purification Kit (NorgenBiotek) in accordance with manufacturer’s protocol. RNA quantification was performed using nanodrop spectrophotometer and purity was determined by 260/280 ratio. The reverse-transcription for total RNA (approximately 1 µg) was performed in final reaction volume of 20 µL containing mixture of 200 units of M-MLV Reverse Transcriptase (Sigma-Aldrich), 10 mM dNTP mix, 5 µM random primers and reaction buffer. The reaction mix was incubated at 37 °C for 50 min followed by 85 °C for 10 min. Reverse transcription for miRNA (~500 ng) was performed with miScript II RT kit (Qiagen) in a final reaction volume of 20 μL that contained 4 μL of Hispec buffer (5×), 2 μL of nucleic mix (10×) and 2 μL miScprit reverse transcriptase. The reaction mix was then incubated at 37 °C for 60 min followed by 90 °C for 5 min.

### Gene expression analysis

All the qPCR experiments for FOS and FTO gene expression were performed in 48-well plates using StepOne Real-Time PCR system (Applied Biosystems) and a predesigned TaqMan gene expression assays for FOS (assay ID: Mm00487425_m1) and FTO (assay ID: Mm00488755_m1) gene (Applied Biosystems). The expression of the FOS and FTO mRNA was normalized to the house keeping gene GAPDH (assay ID: Mm99999915_g1). Thermal cycling was initiated with a denaturation step of 10 min at 95 °C, followed by 45 cycles of 15 s at 95 °C and 90 s at 60 °C. Each sample was analyzed in triplicate alongside with negative control. The data were obtained as cycle threshold (Ct) values and comparative gene expression analysis was performed using and the 2^−ΔΔCt^ relative quantification method.

In silico analysis was performed to establish the relationship between miRNA and FOS and FTO. Three different miRNA prediction softwares including MiRanda (https://www.microrna.org/microrna), Target Scan 7.2 (https://www.targetscan.org/vert_72) and miRwalk (https://zmf.umm.uni-heidelberg.de/apps/zmf/mirwalk2) were used to screen the miRNA-binding possibility against FOS and FTO 3′UTR. Based on their binding potential against FOS and FTO and functional involvement in hypothalamus, a total of twelve miRNAs were selected (listed in Table 1). All the qPCR experiments for miRNA expression were performed in 96-well plates with QuantStudio3 Realtime PCR (Applied Biosystems) using SYBR green (Qiagen). The list of primers used for miRNA expressions is presented in Table 1. The expression of individual miRNA was normalized against the expression of U6SnRNA. Amplification reactions were performed in a volume of 10 µL containing cDNA, SYBR green master mix, corresponding primer assays (forward primer) and a universal primer (reverse primer) for each miRNA. Thermal cycling was initiated with a denaturation step of 10 min at 95 °C, followed by 40 cycles of 15 s at 94 °C and 30 s at 55 °C followed by 30 s 72 °C. Each sample was analyzed in triplicate alongside negative control. The data were obtained as cycle threshold (Ct) values and comparative gene expression analysis was performed using and the 2^−ΔΔCt^ relative quantification method.

**Table 1:** MicroRNA primer sequence

S. no.	miRNA	miRNA primer sequence (5′–3′)
1	mmu-miR-7a-5p	TGGAAGACTAGTGATTTTGTTGT
2	mmu-miR-7b-5p	TGGAAGACTTGTGATTTTGTTG
3	mmu-miR-9-5p For	TCTTTGGTTATCTAGCTGTATGA
4	mmu-miR-29b-3p	TAGCACCATTTGAAATCAGTG
5	mmu-miR-29c-3p	TAGCACCATTTGAAATCGG
6	mmu-miR-30a-5p	TGTAAACATCCTCGACTGGAAG
7	mmu-miR-30b-5p	TGTAAACATCCTACACTCAGC
8	mmu-miR-30c-5p	TGTAAACATCCTACACTCTCAG
9	mmu-miR-338-3p	TCCAGCATCAGTGATTTTGTTG
10	mmu-miR-873a-5p	GCAGGAACTTGTGAGTCTCCT
11	mmu-miR-101b-3p	GTACAGTACTGTGATAGCT
12	mmu-miR-181a-5p	AACATTCAACGCTGTCGGTGAGT
13	mmu-U6SnRNA	TGGCCCCTGCGCAAGGATG

### Protein extraction and western blotting

For western blot analysis, 0.5 × 10^6^ cells were seeded in 100 mm cell culture dish (ThermoFisher Scientific) with different glucose concentrations and were grown for 72 h. Cells were pelleted and washed with ice cold PBS. Protein lysates were prepared by performing cell lysis using M-PER mammalian protein extraction reagent (ThermoFisher Scientific) containing protease inhibitors (ThermoFisher Scientific). Total protein concentrations were measured with standard Bradford method (Bio-Rad). Lysates containing 50 μg of total protein were separated on 12% sodium dodecyl sulfate-polyacrylamide gel electrophoresis (SDS-PAGE) and blotted onto nitrocellulose membrane (Biorad) for 30 min at 30 V in a transfer buffer containing Tris-base and glycine. The membranes were blocked with 5% skim milk prepared in Tris-buffered saline with 0.1% of Tween 20 (TBST) for 1 h and then incubated with primary antibodies against FOS (1:1000 dilutions; Abcam), FTO (1:1000 dilutions; Abcam) and β-actin (1:5000 dilutions; Sigma-Aldrich) at 4 °C overnight. The membranes were then washed with TBST and incubated with horseradish peroxidase-linked secondary antibody at room temperature for 1 h. After TBST washing, the membranes were visualized by clarity western ECL substrate (Bio-Rad) according to the manufacturer’s procedure. The intensity of bands was quantified using image J software.

### MiRNA transfection

1 × 10^5^ cells were cultured in 60 mm cell culture plates (Sigma). Four different hypoglycemic conditions were established by seeding the cells in DMEM containing 2000 mg/L, 900 mg/L, 500 mg/L and 200 mg/L of glucose along with normo-glycemic condition (4500 mg/L). Next day cells were transfected using 60 nM miR-9-5p mimic (Thermo-scientific # 4464066), antimiR-9 (Thermo-scientific # 4464084) and negative control separately with the help of lipofactamine RNAimax (Thermo-scientific). After 24 h, transfected media were replaced by DMEM media containing respective hypoglycemic condition and kept for 72 h. After incubation,  cells were pelleted and washed in ice cold PBS. Protein lysates were prepared by performing cell lysis using M-PER mammalian protein extraction reagent (Thermo scientific) containing protease inhibitors (Thermo scientific) and further continued for western blot analysis.

### Statistical analysis

Data are expressed as ± standard deviation (SD). All statistical analyses were performed using GraphPad Prism 7 for windows (GraphPad Software, CA, USA). Statistical comparisons were performed by one-way analysis of variance (ANOVA) followed by Dunnett’s post hoc test for comparing different groups to control (4500 mg/L glucose) or the Tukey’s correction for multiple comparison. Probability values of *p* < 0.05 were considered statistically significant.

## Results

### Effects of low glucose conditions on the viability of hypothalamic neurons

**Fig. 1:**
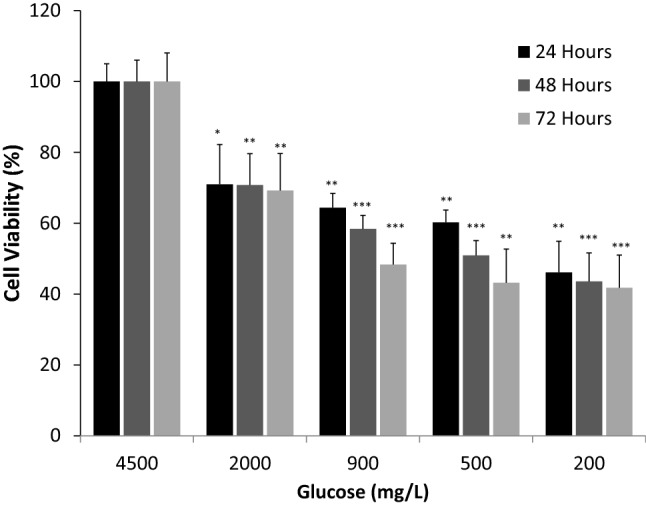
Viability of hypothalamic neurons after exposure to various concentration of glucose. Concentrations of glucose (mg/L) were 4500, 2000, 900, 500 and 200 and the duration of exposure was 24, 48, and 72 h. Data are presented as the mean ± SD (*n* = 6, *P < 0.05; **P < 0.01, ***P < 0.001, *compared to normoglycemia condition of4500 mg/L glucose*)

The first group of experiments was designed to examine the viability of the hypothalamic neurons in response to different conditions of low glucose concentrations. As shown in Fig. 1, the viability of the hypothalamic neurons was decreased significantly due to low glucose conditions and this response was dose dependent (*p* < 0.05). As the concentrations of glucose (2000 mg/L, 900 mg/L, 500 mg/L and 200 mg/L) were reduced, the survival of the neurons was affected, adversely. The duration of exposure to low levels of glucose did not influence the survival of the neurons, significantly. The outcomes of cell viability during 24, 48 and 72 h exposure to low levels of glucose were comparable (Fig. 1). However, the comparison of neurons survival between 24 and 72 h under two conditions with low glucose concentration (900 mg/L and 500 mg/L of glucose), in particular, has shown a significant reduction in the survival rate which was duration dependent (*p* < 0.05).

### Effects of low glucose conditions on the cell death of hypothalamic neurons

**Fig. 2:**
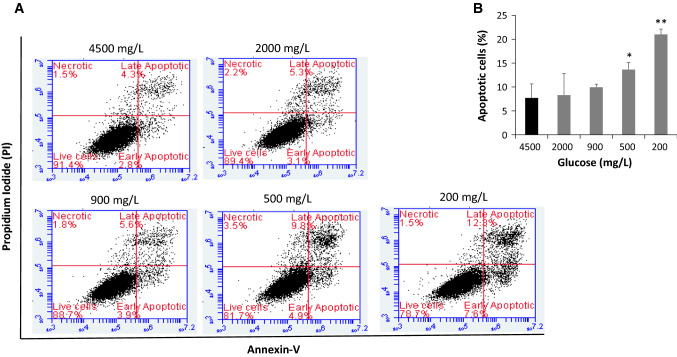
Low glucose condition-induced apoptosis in hypothalamic neurons evaluated by flow cytometry. **a** An increase in the early and late apoptosis of the neurons in a dose-dependent fashion. **b** The rate of apoptosis in the hypothalamic neurons in response to low glucose  conditions (2000–200 mg/L of glucose)

As shown in Fig. 2a, the apoptotic rate was closely related to the glucose concentration and there was a significant shift toward more apoptosis (early and late) in response to conditions with low glucose concentration. This response was dose dependent and the condensation and fragmentation of the nuclei indicating the apoptotic nature, significantly increased as the glucose concentration decreased. Although reduced concentration of glucose produced gradual increase in the apoptotic rate, the two lowest concentrations, 500 and 200 mg/L of glucose, produced the most significant effects (Fig. 2b).

### FOS and FTO mRNA expression in the embryonic hypothalamic neurons in response to low glucose conditions

**Fig. 3:**
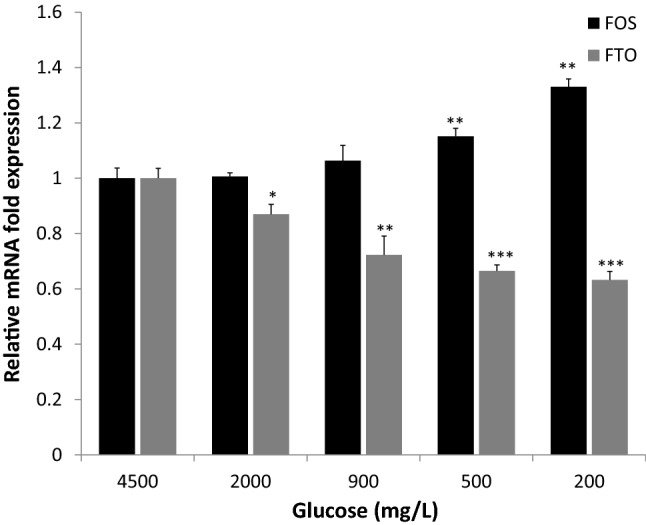
Expression of FOS and FTO mRNA transcripts in the embryonic hypothalamic neurons. Fold difference in FOS (Black Bars) and FTO (Gray Bars) mRNA levels in response to low glucose conditions (2000, 900, 500 and 200 mg/L) was compared with mRNA levels under normoglycemic condition (4500 mg/L) as the  reference = 1, as determined by quantitative reverse transcription-polymerase chain reaction. Error bars represent the standard deviations (*n* = 3, *P < 0.05, **P < 0.01, ***P < 0.001)

Expression of FOS was increased significantly in response to low glucose concentrations and this response was dose dependent (*p* < 0.05; Fig. 3). FOS expression increased as the concentration of glucose decreased indicating an inverse relationship between expression of FOS and low glucose concentration. In contrast, the expression of FTO decreased as the concentrations of glucose were decreased, showing a direct proportional relationship between expression of FTO and glucose concentrations (Fig. 3).

### FOS and FTO protein expression in the embryonic hypothalamic neurons in response to low glucose conditions

**Fig. 4:**
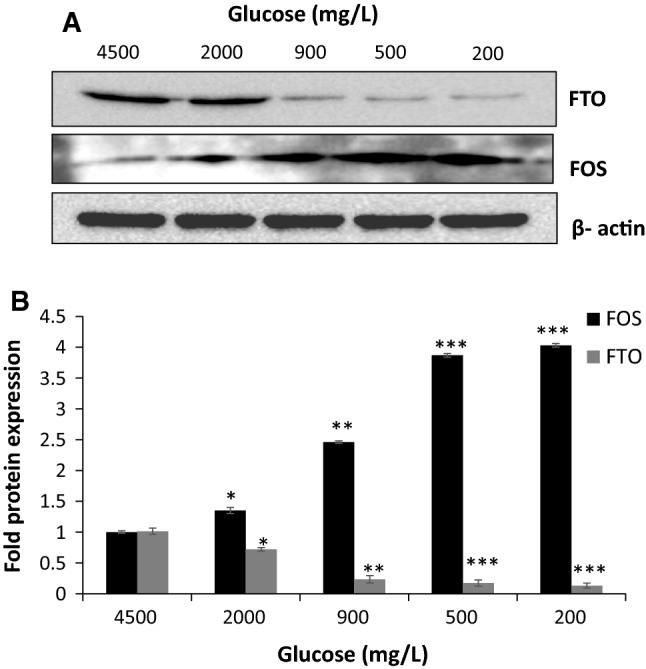
Quantitation of Western blot FOS and FTO protein expression in response to low glucose conditions. **a** Representative Western blot of FOS and FTO in response to different glucose conditions (2000, 900, 500 and 200 mg/L). **b** Protein levels of FOS (Black Bars) and FTO (Gray Bars) were corrected to actin expression (*P < 0.05; **P < 0.01, ***P < 0.001)

As shown in Fig. 4, quantitation of FOS and FTO protein using Western Blotting revealed that FOS expression was increased in response to low glucose conditions (2000 mg/L, 900 mg/L, 500 mg/L and 200 mg/L of glucose) as shown by blots probed with FOS antibody (Fig. 4a, b). In contrast, FTO expression was decreased in response to the same low glucose conditions (Fig. 4a, b). Changes in the expression of both FOS and FTO in response low glucose conditions were statistically significant (*p* < 0.05).

### Differential expression of miRNAs in the embryonic hypothalamic neurons in response to low glucose conditions

**Fig. 5:**
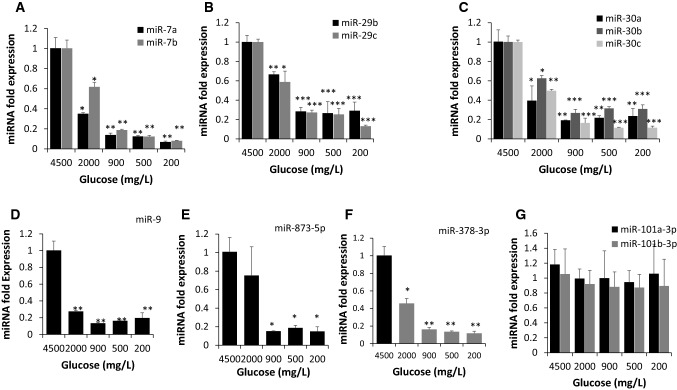
Differential expression of miRNAs in hypothalamic neurons in response to low glucose conditions. **a** Expression of miR-7a (black bar) and -7b (gray bar) was significantly reduced in response to low glucose conditions of 2000, 900, 500 and 200 mg/L (*p < 0.05; **p < 0.01). **b** Expression of miR-29b (Black Bar) and miR-29c (gray bar) was significantly reduced in response to low  glucose  conditions of 2000, 900, 500 and 200 mg/L (*p < 0.05; **p < 0.01; ***p < 0.001). **c** Expression of miR-30a (black bar), -30b (gray bar), -30c (white bar) was significantly reduced in response to low glucose conditions of 2000, 900, 500 and 200 mg/L (*p < 0.05; **p < 0.01; ***p < 0.001). **d** Expression of miR-9 was significantly reduced in response to low glucose conditions of 2000, 900, 500 and 200 mg/L (*p < 0.01). **e**, **f** Expression of miR-378-3p and miR-873-5p was significantly reduced in response to low glucose conditions of 900, 500 and 200 mg/L (*p < 0.05; **p < 0.01; ***p < 0.001). **g** No significant differences were found in the expression of miR-101a-3p and miR-101b-3p under different glycemic conditions (*p* > 0.05)

To investigate the involvement of miRNAs in regulation of FOS and FTO expression under the low glucose conditions, *in silico* analysis was performed using miRanda, Target Scan 7.2 and miRwalk databases. The analysis has shown differential expression of more than 1000 miRNAs with binding potency against FOS and FTO coding sequence and untranslated region (UTR). However, only twelve potential candidates’ miRNAs were identified based on their binding potential against FOS and FTO with a direct involvement in the hypothalamus. The latter includes: miR-7a, miR-7b, miR-9, miR-29b, miR-29c, miR-30a, miR-30b, miR-30c, miR-101b-3p, miR 181a-5p, miR-378-3p and miR-873-5p. As shown in Fig. 5a, the expression of miR-7a, miR-7b was significantly reduced in response to low glucose concentrations of 2000, 900, 500 and 200 mg/L (*p* < 0.05;* p* < 0.01). Expression of miR-29b and miR-29c was significantly reduced in response to low glucose concentrations of 2000, 900, 500 and 200 mg/L (Fig. 5b; *p* < 0.05;* p* < 0.01;* p* < 0.001). Expression of miR-30a, miR-30b, miR-30c was significantly reduced in response to low glucose concentrations of 2000, 900, 500 and 200 mg/L (Fig. 5c; *p* < 0.05;* p* < 0.01;* p* < 0.001). Similarly, the expression of miR-9 was significantly reduced in response to low glucose concentrations of 2000, 900, 500 and 200 mg/L (Fig. 5d; *p* < 0.01). As shown in Fig. 5e, miR- 378-3p was significantly reduced in response to low glucose concentrations of 900, 500 and 200 mg/L (*p* < 0.05). In addition, miR-873-5p was reduced in response to low glucose concentrations of 2000, 900, 500 and 200 mg/L (Fig. 5f; *p* < 0.05;* p* < 0.01). However, no significant change was observed in the expression level of miR-101b-3p and miR-181-5p in response to low glucose conditions (Fig. 5g; *p* > 0.05).

### Regulatory effects of miR-9 on the expression of FOS under low glucose conditions

**Fig. 6:**
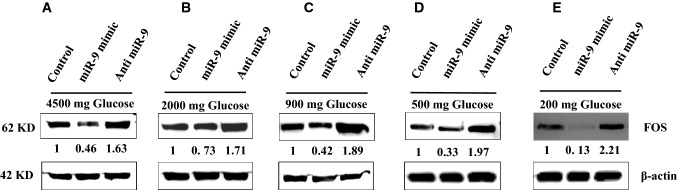
Expression of FOS in response to miR-9 mimic and antimiR-9 using western blot. Representative Western blot of FOS in response to different low glucose conditions in the presence of miR-9 mimic and antimiR-9 (**a** 4500 mg/L glucose, **b** 2000 mg/L glucose, **c** 900 mg/L glucose, **d** 500 mg/L glucose and **e** 200 mg/L glucose)

As mentioned above, twelve potential candidates’ miRNAs were identified based on their binding potential against FOS. Out of these twelve miRNAs, miRNA-9 expression was significantly reduced in response to all conditions of low glucose conditions; therefore, miRNA-9 was chosen as a candidate to do further experiments to assess the role of miRNA-9 in regulation of FOS expression under low glucose concentrations. The results of these experiments have shown that miRNA-9 negatively regulates the expression of FOS. As shown in Fig. 6, FOS expression was decreased in response to reduction of glucose concentration in presence of miR-9 mimic. This response seems to be dependent on the concentration of glucose and, at 2000 mg/L concentration of glucose, the reduction of FOS in the presence of miR-9 mimic was 27% (Fig. 6b), whereas glucose concentration of 200 mg/L resulted in almost 90% reduction (Fig. 6e) in FOS expression in the presence of miR-9 mimic. More importantly, the presence of AntimiR-9 reversed these inhibitory effects of miR-9 mimic and increased the expression of FOS, significantly. This effect seems also to be dependent on the glucose concentration; as in the 2000 mg/L concentration of glucose, the FOS expression increased about 70% (Fig. 6b) in the presence of AntimiR-9 and increased to 120% (Fig. 6e) when the glucose concentration reduced to 200 mg/L.

## Discussion

Hypoglycemia-induced neuronal injury represents a significant challenge to achieve optimal glycemic control in patients with type 1 DM. More importantly, recurrence of hypoglycemic episodes leads to impairment of critical reflexes that are naturally designed to restore normoglycemia and this, in turn, leads to HAAF [13]. Early detection of the latter will improve glycemic control and reduce the serious complications of HAAF. We hypothesized that miRNAs that are involved in regulation of key hypothalamic proteins, such as FOS and FTO, can be clinically relevant biomarkers.

Several neurophysiological studies have investigated different aspects of hypoglycemia and the homeostatic responses to this condition such as CCR [21, 22]. However, none of the previous reports has examined the molecular signature of hypoglycemia in embryonic neurons within the hypothalamus. In addition, the associated effects of hypoglycemia on expression of miRNAs and the hypothalamic regulatory proteins are yet to be reported. The hypothalamus is a heterogeneous region and, therefore, the best model to study the electrophysiological properties of this region is the in vivo model. However, to elucidate the underlying molecular mechanisms of different hypothalamic processes, cell lines represent the most appropriate method and that is why many researchers have turned towards cell lines models [31]. Although primary hypothalamic cultures are heterogeneous and mimic the nature of the hypothalamus in the living brain, they are difficult to maintain and lack viable peptide-secreting neurons. In contrast, embryonic mouse hypothalamus cells (mHypoE-N39) are unlimited homogenous neurons that can be manipulated by various molecular techniques to enrich the molecular aspects of the hypothalamic neurons under different conditions [32]. The latter was the main reason to use these hypothalamic cells in the present study.

The findings of the present study have shown that the survival of the EHNs is dependent on the availability of glucose and, therefore, the hypoglycemic conditions produced a dose-dependent neuronal death. In agreement with these findings, previous reports have shown that hypoglycemia led to more than 90% neuronal death in cortical neuron cultures [33]. This effect was dependent on the time of exposure to glucose where 7 h of glucose deprivation caused more significant neuronal death compared to 3 h [30]. The present study examined the effects of both exposure time and low concentrations of glucose on the survival of the EHNs. Compared to the duration of hypoglycemia, the effects of decreased glucose concentrations seem to be more powerful and consistent. In addition to the different approaches that were used to induce hypoglycemia, the fact that different neurons in the brain possess different thresholds to respond to hypoglycemia explains the variation in the outcomes between the previous and present studies.

Previous electrophysiological studies have used in vitro approach to examine the glucose-sensing behavior within the hypothalamus and this contributed significantly to a better understanding of different aspects of counter-regulation of glycemia and feeding behavior [34, 35]. Most of these studies used a single low dose of glucose concentration to induce hypoglycemia in hypothalamic neurons and the assessment of the neural survival was not the main focus of these studies. On the other hand, the present study has used the approach of gradual reduction of glucose using four doses of low glucose to induce hypoglycemia and also assessed the survival rate of the neurons in more comprehensive manner. It is noteworthy that with recent advancements, the availability of no-glucose media provided an opportunity to prepare the culture medium with lower levels of glucose concentrations and this helped in establishing a better approach to examine the direct effects of hypoglycemia on neural survival [36].

Upon further investigation of the nature of the neuronal death in response to hypoglycemia, findings have shown that the latter induced apoptosis in the EHNs and this was particularly significant at the two lowest concentrations of glucose. These findings were supported by previous in vivo reports which have demonstrated that hypoglycemic rats (1 bout 39 ± 5; 3 bouts 37 ± 4) had more apoptotic cells within the arcuate hypothalamic nucleus cells compared to euglycemic control rats (13 ± 3; *p* = 0.001) [37]**.** These apoptotic effects of hypoglycemia were observed after a single bout of hypoglycemia supporting the hypothesis that apoptosis is the underlying cause of neuronal death in the hypothalamus in response to hypoglycemia.

Although the present study has confirmed the neuronal death in response to direct hypoglycemia, an exceptional phenomenon was observed which is an inverse relationship between FOS expression and glucose concentrations in the EHNs. Previously, changes of FOS expression in response to hypoglycemia were used to determine the location of the neurons that are functionally related to hypoglycemia without in-depth studies about the molecular aspects of FOS expression during the process of neuronal death [38–40]. Although there was no consensus in the outcomes of these previous studies given the heterogeneity of the hypothalamus, the majority suggested that low glucose concentrations lead to reduction in FOS expression [22]. In contrast, the present study has demonstrated that hypoglycemia causes an increase in expression of FOS despite the death of EHNs, suggesting a protective role of FOS against hypoglycemia. This may reflect the original function of FOS since the present experiments were conducted in embryonic neurons whereas all previous experiments were performed in adult neurons. On the other hand, two categories of neurons were previously suggested based on the behavior of glucose sensing: (i) glucose responsive (GR) excited neurons and (ii) glucose-sensitive (GS) inhibited neurons [41]. In the latter, it is natural that lowering glucose increases FOS expression, cytosolic Ca^2+^ and membrane depolarization and these parameters were used as markers for neuronal excitation of GS neurons in the lateral hypothalamic areas [42, 43] and the arcuate nucleus [44, 45]. To consider these previous findings as alternative explanation for our present studies, we believe that further studies need to be conducted to investigate the electrophysiological features of EHNs under hypoglycemic conditions.

Simultaneously, FTO expression was assessed in the same hypoglycemic environment. Interestingly, reduced hypothalamic FTO expression was reported in response to hypoglycemia in a dose-dependent fashion. In agreement with this finding, a study by Poritsano et al. has shown that fasting negatively influences FTO mRNA levels and the number of FTO-immunoreactive cells in the hypothalamus. These outcomes were reversed in response to administration of glucose [2]. In the light of these findings and along with the present outcomes, we can confirm the regulatory role of hypothalamic FTO in the mechanisms of hypoglycemia in adult and embryonic neurons within the hypothalamus. In addition, an interesting relationship between variants in FTO and obesity has been suggested emphasizing the role of FTO in central regulation of several metabolic processes [46]. This was further supported by the findings that mouse models with the absence of FTO exhibited reduced body weight whereas overexpression of FTO resulted in an increase in body weight [47, 48]. Although FTO is expressed in various tissues, its level is particularly high in the hypothalamus and this suggests that the hypothalamic FTO plays an important role in glucose metabolism as well as in obesity. Given that the obesity has a strong heritable component, further genetic studies have shown that FTO encodes for an enzyme able to remove methyl groups from DNA and RNA nucleotides in vitro and human studies, emphasizing the involvement of epigenetic mechanisms in obesity [49, 50]. Taking into account the findings of the previous and present studies, we can suggest an association between hypothalamic FTO and epigenetic alterations that may involve in hypoglycemia and obesity; however, further studies are required to support this suggestion.

Recently, the involvement of miRNAs in regulation of hypothalamic functions has received much attention. Several genes within the hypothalamus are regulated by miRNAs and, therefore, it is not surprising that these non-coding RNAs are potential biomarkers and modulators for various diseases and future therapeutic targets [14, 51–53]. Given the holistic role of hypothalamus in regulation of several physiological functions including food intake and body weight, previous reports have investigated the involvement of miRNAs in these functions [14]. However, the involvement of miRNAs in detection or responding to glycemic changes is considered as a novel field of research. In the present study, screening for miRNAs has shown that more than 1000 miRNAs are differentially expressed in the hypothalamus. Nevertheless, overlapping binding sites for twelve miRNAs were identified in FOS and FTO. It is noteworthy that there was an overlap in the miRNAs between FTO and FOS despite the fact that both had differential responses to hypoglycemia. This highlights an important interrelationship between central integration of food intake, obesity and glycemic control.

In addition, these inhibitory regulators were gradually suppressed by reduction in glucose concentrations suggesting that hypoglycemia may have negative effect on the expression of these miRNAs. Taking into account this finding, we can suggest that the noticeable increase in FOS expression in response to hypoglycemia may result from the inhibition of miRNAs expression. This suggestion was strongly supported by the finding that miR-9 negatively regulates the expression of FOS and this effect was dependent on the concentration of glucose. As the latter decreases, the miR-9 mimic produces more significant reduction in the expression of FOS and AntimiR-9 leads to pronounced increase in expression of FOS. We believe that the relationship between hypothalamic miRNAs and regulatory proteins, FOS and FTO, builds a strong potential for identification of novel biomarkers for HAAF.

## Conclusion

The results of the present study demonstrated that optimal glycemic level is crucial for survival of EHNs. FOS expression was significantly increased in response to hypoglycemia suggesting a protective role in this condition. Twelve miRNAs that are related to both FOS and FTO were identified and the expression of ten of them was reduced significantly in response to hypoglycemia. This interrelationship between the hypothalamic miRNAs and the regulatory protein, FOS and FTO, in response to hypoglycemia facilitates an opportunity to identify potential biomarkers and novel therapeutic targets for HAAF.

## Abbreviations

CRR: Counter-regulatory response
DM: Diabetes mellitus
FTO: Fat mass and obesity-associated gene/protein
HAAF: Hypoglycemia-associated autonomic failure
ENHs: Embryonic hypothalamic neurons
